# Louisiana

**Published:** 1896-08

**Authors:** 


*Louisiana.* Charbon has broken out in enzoötic form among the mules of northern Louisiana. Pasteur vaccine is being extensively employed to control its spread.

